# Xoo-YOLO: a detection method for wild rice bacterial blight in the field from the perspective of unmanned aerial vehicles

**DOI:** 10.3389/fpls.2023.1256545

**Published:** 2023-10-23

**Authors:** Pan Pan, Wenlong Guo, Xiaoming Zheng, Lin Hu, Guomin Zhou, Jianhua Zhang

**Affiliations:** ^1^ National Agriculture Science Data Center, Agricultural Information Institute, Chinese Academy of Agricultural Sciences, Beijing, China; ^2^ National Nanfan Research Institute (Sanya), Chinese Academy of Agricultural Sciences, Sanya, China; ^3^ Institute of Crop Science, Chinese Academy of Agricultural Sciences, Beijing, China; ^4^ Farmland Irrigation Research Institute, Chinese Academy of Agricultural Sciences, Xinxiang, China

**Keywords:** wild rice, UAV, bacterial blight, disease detection, deep learning, YOLOv8

## Abstract

Wild rice, a natural gene pool for rice germplasm innovation and variety improvement, holds immense value in rice breeding due to its disease-resistance genes. Traditional disease resistance identification in wild rice heavily relies on labor-intensive and subjective manual methods, posing significant challenges for large-scale identification. The fusion of unmanned aerial vehicles (UAVs) and deep learning is emerging as a novel trend in intelligent disease resistance identification. Detecting diseases in field conditions is critical in intelligent disease resistance identification. In pursuit of detecting bacterial blight in wild rice within natural field conditions, this study presents the Xoo-YOLO model, a modification of the YOLOv8 model tailored for this purpose. The Xoo-YOLO model incorporates the Large Selective Kernel Network (LSKNet) into its backbone network, allowing for more effective disease detection from the perspective of UAVs. This is achieved by dynamically adjusting its large spatial receptive field. Concurrently, the neck network receives enhancements by integrating the GSConv hybrid convolution module. This addition serves to reduce both the amount of calculation and parameters. To tackle the issue of disease appearing elongated and rotated when viewed from a UAV perspective, we incorporated a rotational angle (theta dimension) into the head layer's output. This enhancement enables precise detection of bacterial blight in any direction in wild rice. The experimental results highlight the effectiveness of our proposed Xoo-YOLO model, boasting a remarkable mean average precision (mAP) of 94.95%. This outperforms other models, underscoring its superiority. Our model strikes a harmonious balance between accuracy and speed in disease detection. It is a technical cornerstone, facilitating the intelligent identification of disease resistance in wild rice on a large scale.

## 1 Introduction

Wild rice, which is closely related to cultivated Asian rice (referred to as “rice” hereafter), serves as a valuable parental material for rice breeding. It shares the same chromosome group as rice and harbors exceptional genes that are either absent or lost in rice. Consequently, wild rice acts as a fundamental germplasm resource for genetic enhancement in rice (Yuan, 1986; Fan et al., 2023; Shao et al., 2023). As the genetic background for disease resistance in rice becomes increasingly limited, the screening of superior disease resistance genes in wild rice, which have been lost during the domestication of rice, and utilizing them in breeding emerges as an effective and cost-efficient approach to mitigating rice diseases. The identification of disease resistance in wild rice is one of the key aspects of breeding disease-resistant varieties of rice. In the process of identifying new genes with broad-spectrum and durable disease resistance traits and resolving their molecular mechanisms of resistance, different wild rice resources need to be identified and validated for disease resistance in order to select high-quality germplasm resources and breeding materials with high disease resistance. The identification of disease resistance in wild rice is instrumental in bolstering the construction, conservation, and utilization of wild rice germplasm resources, as well as in breeding superior rice varieties with enhanced disease resistance (Yun and Han, 2014).

Rice bacterial blight, caused by *Xanthomonas oryzae* pv. *oryzae*, is a highly detrimental bacterial disease that significantly affects the growth of rice. This disease not only leads to a decrease in yield but also negatively impacts the quality of rice (Chen et al., 2021). In comparison to the utilization of fungicides, the utilization of bacterial blight resistance genes from wild rice and the development of genetic varieties with broad-spectrum resistance to bacterial blight offer economic, environmentally friendly, and safe alternatives. This approach is currently a prominent research focus in the field of plant immunity and a crucial objective for crop breeding worldwide (Xu et al., 2019). In the conventional process of disease resistance identification, researchers are required to visually observe and manually test the presence of diseases in wild rice within relatively distant experimental fields, which is, however, both time-consuming and subjective. Given the growing demand for large-scale identification of disease resistance in wild rice, there is a pressing need to develop a high-throughput and intelligent approach for accurately identifying disease resistance in wild rice on a large scale, which would enable automated disease resistance identification in the field.

The method of field-based wild rice disease detection enables precise localization of diseases affecting wild rice from an unmanned aerial vehicle (UAV) perspective. It serves as a crucial component in the intelligent identification of wild rice disease resistance, providing fundamental support for post-processing tasks such as wild rice disease segmentation, disease spot measurement, and disease resistance identification. Deep learning algorithms possess the ability to autonomously learn and represent features, and they can partially replace manual disease detection with their high robustness and accuracy (Liu and Wang, 2021; Shao et al., 2022). It has been extensively utilized in the detection of diseases in maize (Khan et al., 2023), potatoes (Dai et al., 2022), strawberries (Lee et al., 2022), citrus (Qiu et al., 2022), and other crops (Dai and Fan, 2022). In recent years, researchers have employed deep learning techniques to detect rice bacterial blight. For instance, Jia et al. (2023) utilized MobileNetV3 to substitute the original YOLOv7 algorithm’s backbone network. They integrated the coordinate attention (CA) module into the feature fusion layer of YOLOv7 to impart richer semantic information. Additionally, they incorporated SIoU to bolster precision and robustness, mitigating overfitting concerns. This enhancement led to an impressive average precision (AP) of 98%. To address the challenges associated with the undefined value of “k” in the k-means clustering algorithm, which often leads to suboptimal solutions, Zhou et al. (2019) proposed the FCM-KM algorithm. This innovative approach employs maximum and minimum distances to determine both the optimal “k” value and the central positions for clustering. To enhance the detection of rice disease, they integrated the FCM-KM algorithm with the Faster R-CNN model. This fusion yielded impressive results, with a detection accuracy of 97.53% and a processing time of 0.62 s. In a similar vein, Prasomphan (2023) utilized the YOLOv3 model, achieving a notable AP of 89.6%. Kumar et al. (2023) introduced a multi-scale YOLOv5 detection network. This innovation in detection accuracy is achieved through the integration of the DAIS segmentation and Bi-FAPN networks. Their approach also effectively reduces computational costs by employing the principled pruning technique. Remarkably, their model achieves a mAP of 82.8% on the RLD dataset. On the other hand, Haque et al. (2022) employed the YOLOv5 model, attaining an AP of 65%.

The efforts of the aforementioned researchers have certainly propelled the progress of rice bacterial blight detection. They have contributed valuable insights in areas such as dataset enhancement and optimization of detection algorithms. However, there remains room for further improvement in accurately detecting small and densely clustered targets within the intricate field conditions. Moreover, considering various angles of the disease under the UAV viewpoint and acknowledging the subtle disparities in visual features between wild and cultivated rice diseases, the current methodologies are inadequate to meet the demands of disease-resistant breeding applications for wild rice bacterial blight. Furthermore, in the realm of object detection models, achieving real-time wild rice bacterial blight detection from UAV necessitates the integration of algorithms with swift inference capabilities. The faster R-CNN model (Ren et al., 2017), representing a two-stage detection model, demonstrates higher accuracy but slower speed. On the other hand, the YOLO model, representing a one-stage detection model, offers a significant advantage in speed compared to the two-stage model, making it better suited for real-time detection requirements (Pan et al). Hence, this study opts for the latest YOLO series algorithm, YOLOv8, as the baseline model.

With a specific focus on wild rice bacterial blight, the research introduces a novel approach built on the Xoo-YOLO model for detecting bacterial blight in wild rice from the viewpoint of UAV within field environments. This method is designed to address challenges associated with low accuracy in detection under the perspective of UAV in fields. It also addresses the inability of the horizontal bounding box detection to detect various angles of the disease detected from the UAV viewpoint. The overarching aim is to enhance the precision and robustness of bacterial blight detection in wild rice when viewed from the UAV’s perspectives within field conditions.

The important contributions of this paper are as follows:

(1) Images of wild rice bacterial blight were collected as a dataset from the perspective of UAV in natural field environments. These images were utilized for training, validating, and testing the model.
(2) A method for wild rice bacterial blight detection in the field from the UAV perspective, which is based on the Xoo-YOLO model, is proposed to address the issue of poor detection performance for dense or small-object disease targets and balancing both the detection accuracy and speed. This method meets the demand for wild rice bacterial blight detection in breeding disease resistance. The backbone network was introduced into the Large Selective Kernel Network (LSKNet) to better achieve the detection of disease targets under the UAV viewpoint by dynamically adjusting its large spatial receptive field. Simultaneously, the neck network is enhanced by introducing the hybrid convolution module of the GSConv to reduce the amount of calculation and parameters of the model.
(3) An oriented bounding box detection method with rotating angles (theta dimension) is proposed to address the issue of inaccurate detection brought on by the wild rice bacterial blight, which presents arbitrary angles from the UAV viewpoint. This method achieves localized detection of wild rice disease spots in arbitrary directions while reducing the interference brought about by too much background information introduced and improving the network’s ability to extract disease.
(4) Through experiments, the proposed Xoo-YOLO model has been validated on the dataset of wild rice bacterial blight. Across metrics such as accuracy, recall, and *F*1 score, this approach consistently outperforms. Notably, it achieves this superiority while effectively reducing model parameters and computational complexity. Striking a balance between accuracy and efficiency, it is better poised to cater to real-time detection requirements under resource-constrained settings, such as UAV and other edge devices.

## 2 Materials and methods

### 2.1 Materials

#### 2.1.1 Material preparation

Wild rice germplasm resources from the Institute of Crop Science, Chinese Academy of Agricultural Sciences, were planted at the Potianyang Base, Yazhou District, Sanya City, Hainan Province, China (N: 18°39′84.13″, E: 109°17′51.68″). On 1 March 2023, a total of 120 wild rice samples were artificially inoculated with the pathogen of rice bacterial blight. Following the “The technique rules for identification of rice variety resistance against bacterial blight (*Xanthomonas oryzae* pv. *oryzae*),” the inoculation was performed using the pathogenic strain PXO99A of *Xanthomonas oryzae* pv. *oryzae*, which is known to cause rice bacterial blight. The inoculation method involved manually cutting leaves during the tillering stage (Administration, A.P.M.S, 2017; Tang et al., 2017).

#### 2.1.2 Image acquisition and processing

**Figure 1:**
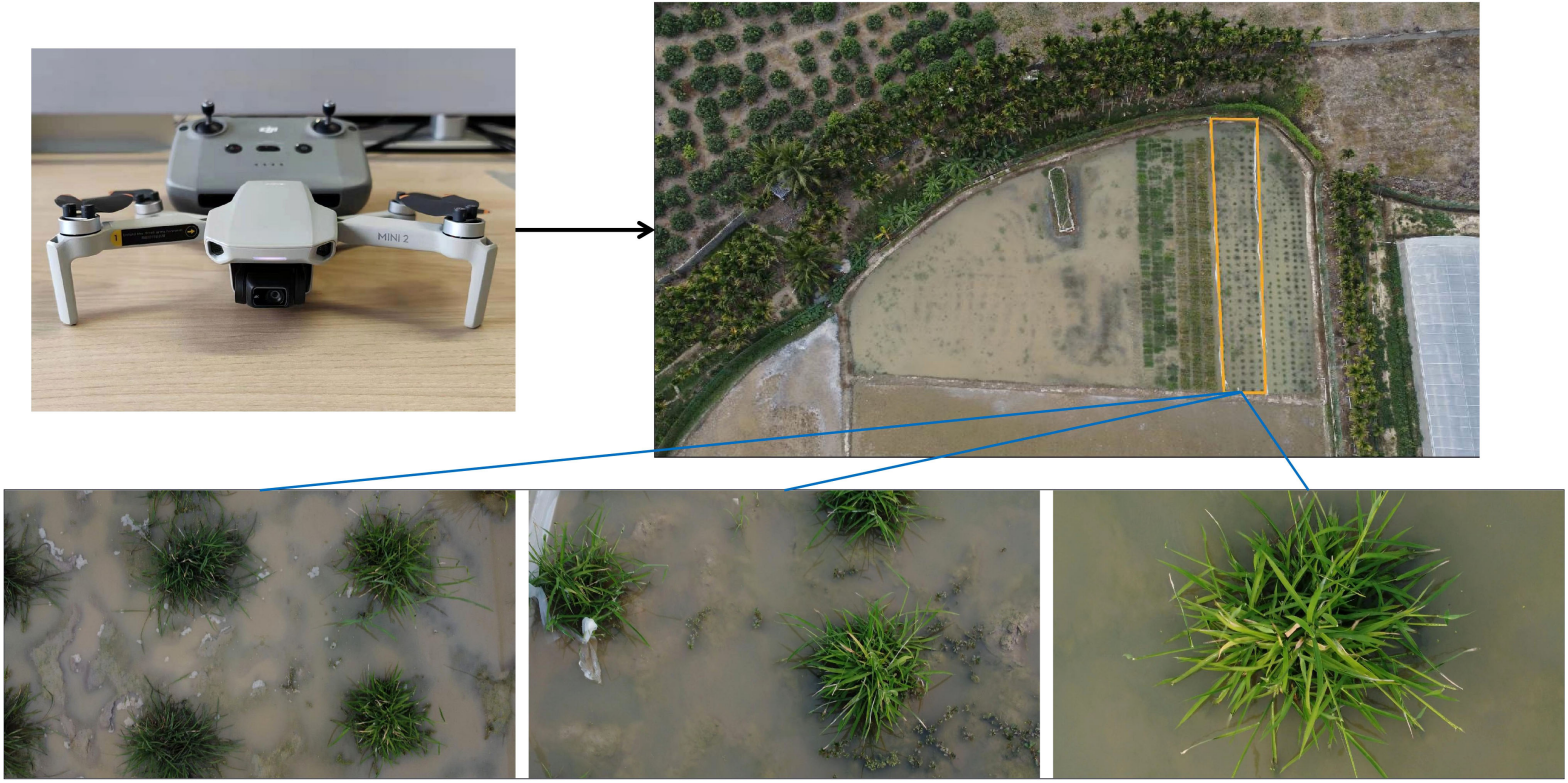
Using an unmanned aerial vehicle (UAV) to acquire image data of wild rice infected with bacterial blight.

**Figure 2:**
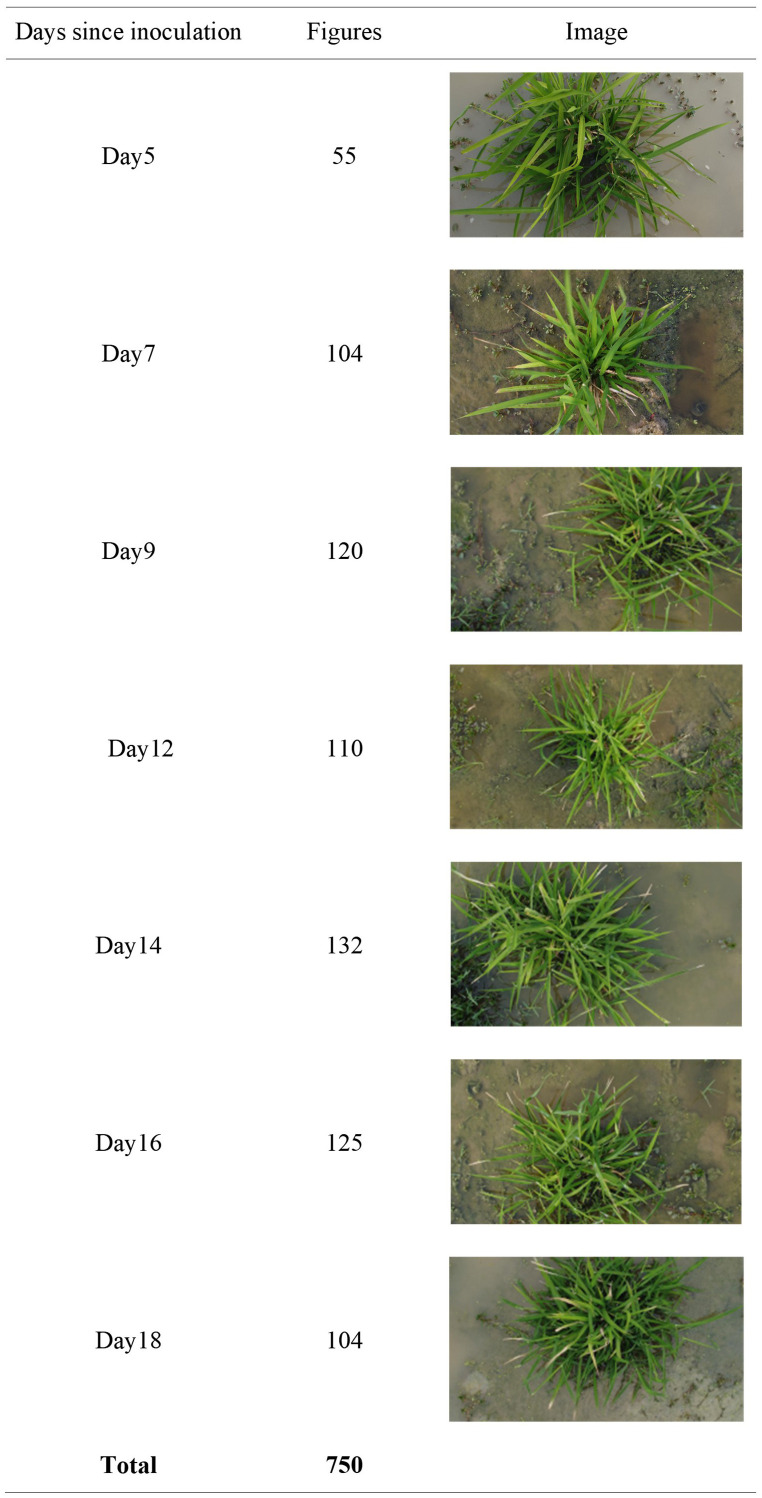
The days since inoculation, figures, and image samples in the wild rice bacterial blight dataset.

The camera-equipped UAV was utilized to acquire RGB images of wild rice at different times after infection with bacterial blight. The image acquisition method of wild rice bacterial blight is shown in **Figure 1**. The type of UAV equipment is Dji Mini2 (DJI Inc, Shenzhen, China), the flying altitude is 0.6–1.5 m above the surface of the wild rice field, the camera head pitch angle is −90° to −60°, and the output image resolution is 1,920 × 1,080, which is saved in JPG format. Image acquisition was conducted at different time intervals, specifically on the fifth, seventh, ninth, 12th, 14th, 16th, and 18th days after infection. The acquisition periods were from 8:00 AM to 11:00 AM and from 4:30 PM to 6:30 PM. The weather conditions during image acquisition included sunny, cloudy, and overcast days, with environmental temperatures ranging from 22°C to 30°C. All images were captured in the natural field environment, utilizing natural lighting without the use of flash. The images contained various types of interference, such as different levels of occlusion, water surface reflections, and overexposure, as well as weeds, withered leaves, bird droppings, and field debris. Each image contained one-fourth to two wild rice plants. In total, 750 images of infected wild rice with bacterial blight were collected under the UAV viewpoint. The days since inoculation, figures, and image samples in the wild rice bacterial blight dataset are shown in **Figure 2**. These images were further identified and confirmed by two experts specializing in wild rice germplasm identification. The open-source software roLabelImg (cgvict, 2017) was used to manually annotate wild rice leaf blight spot areas, and the annotation information was saved as a file in.xml format using this software. The dataset was divided and split in the ratio of 6:2:2, with 450 images randomly selected as the training set, 150 as the validation set, and 150 as the test set. Furthermore, the inclusion of mosaic augmentation is notable during training. Mosaic augmentation involves the random extraction of four images, where each image contributes only a portion of its content and corresponding detection box information. These fragments are then combined into a single image, serving as input for the network. This technique significantly diversifies the training data, effectively guarding against overfitting by introducing greater variability into the learning process.

### 2.2 Methods

#### 2.2.1 Overall model

**Figure 3:**
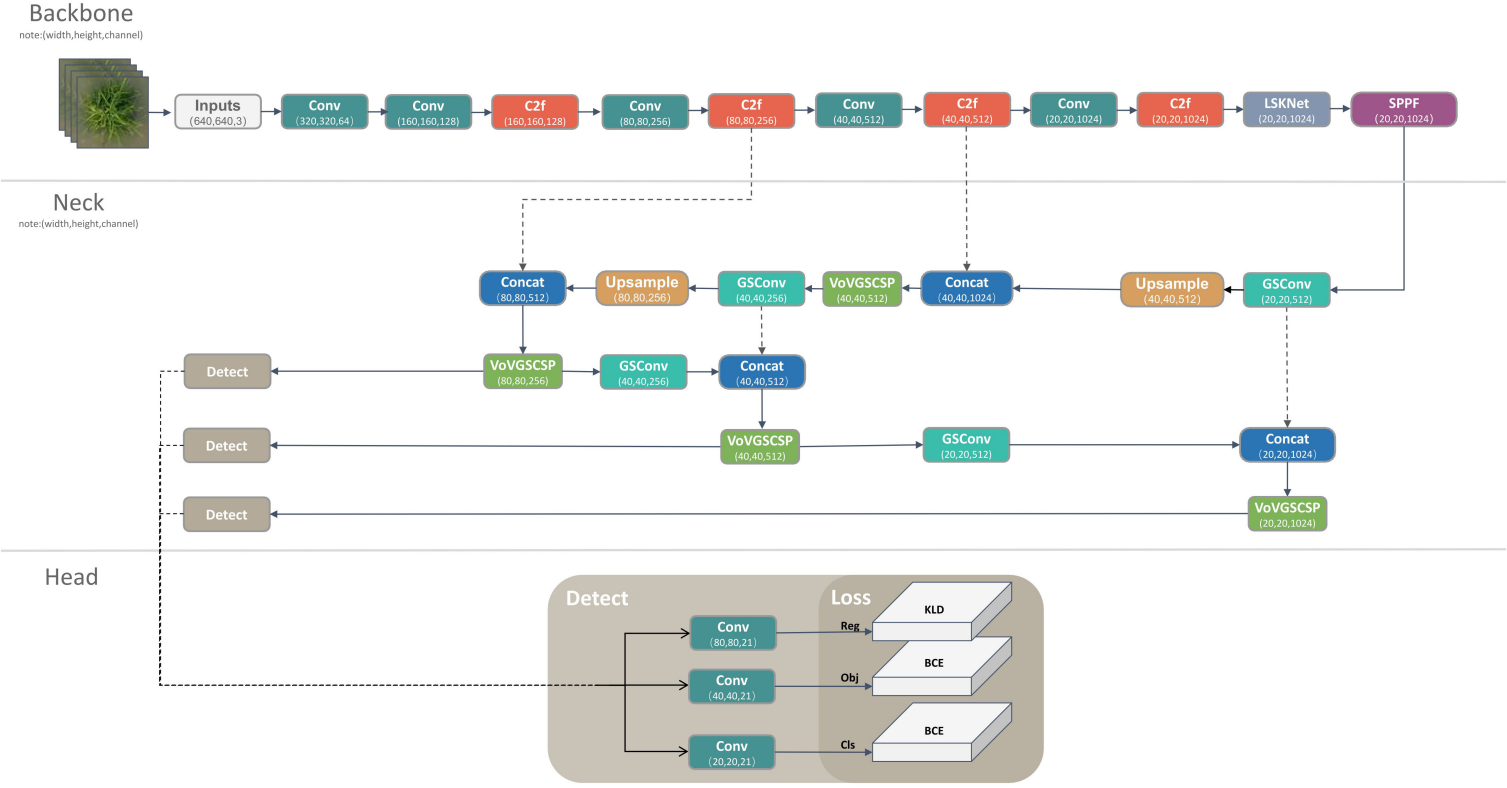
Overall model architecture diagram.

Given the intricate backgrounds inherent in wild rice paddies—encompassing water, shadows, and reflections—and the dynamic alterations in the manifestation of visual disease attributes due to the interplay of UAV propeller airflow and variable weather conditions, the imperative for real-time detection underscores our decision to employ YOLOv8 as the baseline model for this research. In this context, Xoo-YOLO emerges as an enhanced iteration built upon YOLOv8, specifically tailored for the task of detecting wild rice bacterial blight from the UAV perspective in the field. The architecture of Xoo-YOLO is visualized in **Figure 3**.

The model comprises four principal components: input, backbone, neck, and head. The enhancements incorporated are outlined as follows:

(1) Integration of LSKNet into the backbone: To better achieve the detection of disease targets under the UAV viewpoint, we introduced LSKNet into the backbone network, which adopts the dynamic adjustment of its large spatial receptive field, allowing the model to adaptively use different large kernels and adjust the receptive field for each target in space as needed.
(2) Construction of a lightweight model: In pursuit of a more streamlined design, the neck network is enhanced by introducing the hybrid convolution module of the GSConv to reduce the number of calculations and parameters of the model.
(3) Inclusion of an oriented bounding box detection method: To detect the wild rice bacterial blight under the UAV viewpoint with arbitrary angles, we introduce an oriented bounding box detection method. This approach integrates angle information (theta) within the head layer and loss function, reducing the interference brought about by too much background information and improving the network’s ability to extract disease.

By amalgamating these advancements, Xoo-YOLO is equipped to address the intricacies associated with wild rice bacterial blight detection from the UAV viewpoint, offering improved accuracy and robustness. The whole algorithm is summarized in the pseudo-lcode in **Algorithm 1**.

**Algorithm 1:** Pseudocode of Xoo-YOLO Algorithm.

**Input:** Image I, Confidence Threshold T **Output:** Detected objects with oriented bounding boxes and labels 1. Scaling and normalizing the input image I 2. Feed image I into the network to obtain the output feature map 3. Prediction for each grid unit using the feature map: a. Predict the presence of an object in each grid unit using the BCE function b. Predict the category of objects in each grid unit using the BCE function c. Predict the location and size of objects in each grid unit using the KLD function 4. Post-processing of predicted bounding boxes: a. Remove bounding boxes with confidence lower than threshold T b. Apply non-maximum suppression to remove overlapping bounding boxes 5. Output final prediction results: Category, confidence, and location information of wild rice bacterial blight

#### 2.2.2 YOLOv8

YOLOv8, a prominent object detection algorithm, was introduced by Ultralytics in January 2023. This algorithm has exhibited commendable outcomes in both speed and accuracy (Terven and Cordova-Esparza, 2023). The backbone of YOLOv8 centers around the C2f module, drawing inspiration from the ELAN module. The C2f module is constructed from two Conv modules and multiple Darknet BottleNeck modules, interconnected by Split and Concat modules. Additionally, YOLOv8 incorporates the Spatial Pyramid Pool Fusion (SPPF) module. This innovation transforms feature maps of varying sizes into fixed-size feature vectors, effectively preserving the original image’s features and positional information to the maximum extent. The YOLOv8’s neck network incorporates the Path Aggregation Network (PANet), similar to YOLOv7 (Wang et al., 2023), which fuses three effective feature layers obtained from the backbone network across layers of features, with the three effective feature layers located in the middle, lower middle, and bottom layers of the backbone network.

#### 2.2.3 Improved backbone network structure

Owing to the intricate setting of wild rice paddies, encompassing water, mud, algae, weeds, bird droppings, shadows, and other intricate backgrounds, coupled with the relatively diminutive size of wild rice bacterial blight as viewed from UAV, relying on limited contextual information often triggers incorrect detection. For instance, it may lead to the misinterpretation of white streaks of debris within the wild rice paddies as instances of wild rice bacterial blight. Simultaneously, the varying viewpoints and distances of the UAV introduce distinct contextual information requirements for accurate detection. However, introducing an excess of contextual information can inadvertently obscure target features’ specifics while exacerbating the model’s complexity.

To address the aforementioned challenges, the presented Xoo-YOLO model incorporates the LSKNet (Li et al., 2023). LSKNet dynamically adjusts its large spatial receptive field, enabling the model to flexibly employ varying large kernels and modify the receptive field according to the specific spatial requirements of each target. This adaptability is especially vital for detecting wild rice bacterial blight amid the UAV viewpoint and intricate conditions of the field.

LSKNet is implemented through a spatial selection mechanism, which is implemented by effectively weighting the features processed by a sequence of large depth-wise convolutional kernels and spatially merging them. The weights of this kernel are dynamically determined based on the input, allowing the model to adaptively use different large kernels and adjust the receptive field for each target in space as needed.

LSKNet mainly consists of two sub-blocks, Large Kernel Selection (LK Selection) and Feed-Forward Network (FFN). FFN is used for channel mixing and feature refinement and consists of a sequence of a fully connected layer, a depth-wise convolution, a GELU activation, and another fully connected layer; LK Selection consists of a fully connected layer, LSK sub-block, a GELU activation, and another fully connected layer.

The central component of LSKNet is the LSK sub-block, which comprises a series of large kernel convolutions alongside a spatial kernel selection mechanism. Larger kernel convolution constructs by explicitly decomposing it into a sequence of depthwise convolutions with a large growing kernel and increasing dilation. To elaborate, the expansion of various parameters, such as kernel size “*k*,” dilation rate “*d*,” and the receptive field “RF,” within the *i*th depthwise convolution within the sequence, is characterized by the following definitions:


(1) $$\begin{array}{l} k_{i-1}\leq k_{i};d_{1}=1,d_{i-1}<d_{i}\leq {\text{RF}}_{i-1},\\ {\text{RF}}_{1}=k_{1},{\text{RF}}_{\text{i}}=d_{i}\left(k_{i}-1\right)+{\text{RF}}_{i-1}. \end{array}$$

The increasing kernel size and dilation rate ensure that the receptive field expands quickly enough, and at the same time, an upper bound on the dilation rate is set to avoid the dilation convolution introducing gaps between feature maps. This approach makes the later kernel selection easier and also reduces the number of parameters significantly. Simultaneously, a series of decomposed depthwise convolutions with different receptive fields are used to obtain features with contextual information at different ranges, allowing channel blending for each spatial feature vector. The calculation is shown in Eqs. (2) and (3).


(2) $$U_{0}=X,U_{i+1}={ℱ}_{i}^{dw}\left(U_{i}\right)$$

(3) $${\tilde{U}}_{i}={ℱ}_{i}^{1\times 1}\left(U_{i}\right),\text{ for }i\text{ in }\left[1,N\right]$$

Based on the above, LSKNet employs a spatial kernel selection mechanism to enhance the network’s ability to focus on the most relevant spatial context regions, spatially selecting feature mappings from large convolutional kernels of different scales.

The outlined implementation steps are as follows:

(1) Concatenate features obtained from different kernels with different ranges of receptive fields:


(4) $$\tilde{U}=\left[{\tilde{U}}_{1};\ldots ;{\tilde{U}}_{i}\right]$$

(2) Efficient spatial relation extraction using channel-based average pooling and maximum pooling: 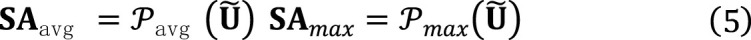


ally pooled features and use a convolutional layer ${ℱ}^{2\to N}$ (·) to transform the pooled features (with two channels) into *N* spatial attention maps:


(6) $$\hat{SA}={ℱ}^{2\to N}\left(\left[SA_{\text{avg }};SA_{\text{max}}\right]\right)$$

(4) A sigmoid activation function is used to obtain a separate spatial selection mask for each decomposed kernel:


(7) $$\tilde{\text{S}{\text{A}}_{i}}=\sigma \left({\hat{SA}}_{i}\right)$$

(5) The features in the decomposed macronucleus sequence are weighted with the corresponding spatially selective masks and fused by a convolutional layer ℱ (·) to obtain the attention feature ***S***:


(8) $$S=ℱ\left(\sum_{i=1}^{N}\left(\tilde{\text{S}{\text{A}}_{i}}\cdot {\tilde{\text{U}}}_{i}\right)\right)$$

(6) The final output of the LSK module is an element-by-element product between input features *X* and *S*:


(9) Y=X·S

**Figure 4:**
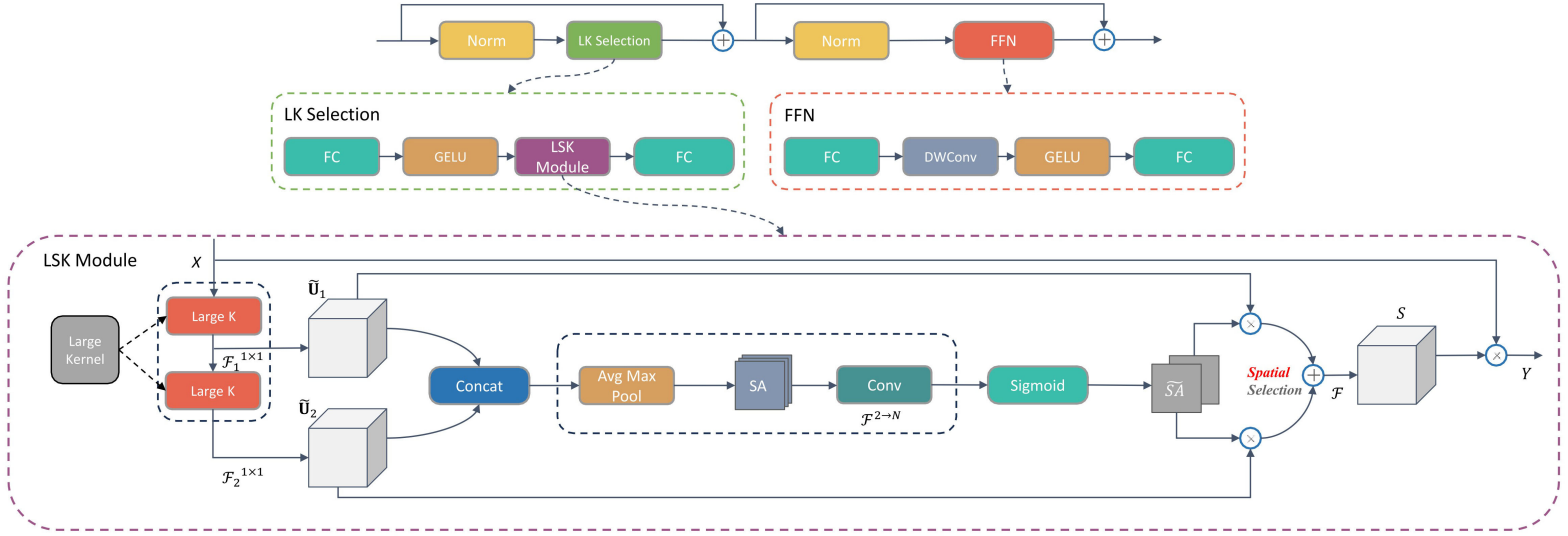
Structural diagram of LSKNet.

LSKNet fulfills the requirement for detecting bacterial blight in wild rice from the perspective of UAV in the field while addressing the necessity for a wider and adaptable contextual understanding without bells and whistles. Therefore, we add LSKNet to the backbone network to enhance the feature extraction capability of this model. The structural diagram of LSKNet is illustrated in **Figure 4**.

#### 2.2.4 Improved neck network structure

The standard convolution (SC) module used in YOLOv8 applies different convolutional kernels to multiple channels simultaneously, which leads to an increase in the number of parameters required and high FLOP. On the other hand, although lightweight networks using depthwise separable convolutions (DSC) such as MobileNet (Howard et al., 2017) and ShuffleNet (Zhang et al., 2018) can effectively solve this problem and greatly improve the detection performance, DSC separates channel information from the input image during computation, leading to a significant reduction in the feature extraction and fusion capabilities and resulting in a decrease in the detection performance of the model. It cannot meet the real-time requirements of disease resistance identification for the detection of wild rice bacterial blight.

To enhance computation speed without compromising detection accuracy, the Xoo-YOLO model integrates the GSConv hybrid convolution module (Li et al., 2022). This module incorporates Shuffle to infuse information produced by SC into the information generated by DSC. In contrast to DSC, the strength of GSConv lies in its ability to maintain hidden connections while operating with reduced complexity. This approach adeptly strikes a model equilibrium between accuracy and speed, ensuring a judicious trade-off between the two.

**Figure 5:**
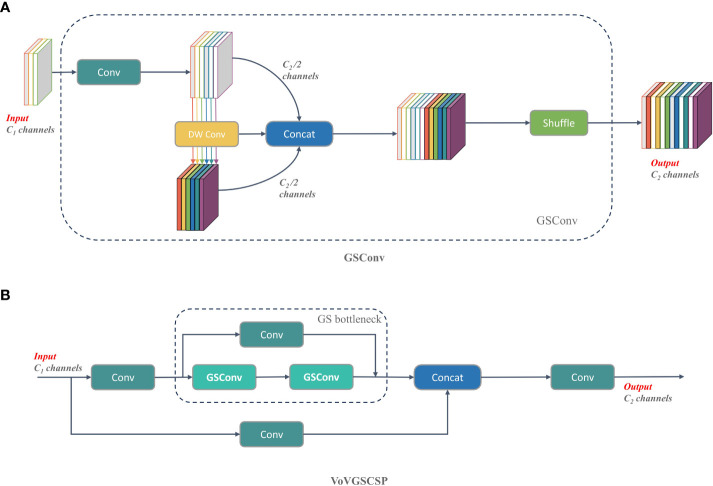
Structural diagram of GSConv and VoVGSCSP module. **(A)** GSConv module. **(B)** VoVGSCSP module.

The GSconv module is primarily composed of Conv, DWConv, Concat, and Shuffle operations. This configuration is illustrated in **Figure 5A** and is constructed as follows:

(1) The input feature map has a total of C1 channels.
(2) DSC is applied to half of the channels, while SC is applied to the remaining half.
(3) The resulting two output feature maps are concatenated along the channel dimension.
(4) The concatenated feature map is then subjected to a shuffle operation, yielding the final output.
(5) The final output feature map possesses a total of C2 channels.

VoVGSCSP is an iterative integration of the GS bottleneck utilizing the foundation of GSConv (Xu et al., 2023). The process involves segmenting the input feature map’s channel count into two segments. The initial portion traverses through convolution (Conv) for processing, following which the features undergo extraction through consecutively stacked GS bottleneck modules. On the other hand, the remaining segment is utilized as residuals, engaging in a single convolution operation. This module aptly harmonizes the model’s accuracy and speed, resulting in a reduction of both computational and complexity. Simultaneously, it sustains a commendable degree of accuracy and significantly augments the reutilization rate of extracted features. The structural representation of VoVGSCSP can be observed in the diagram depicted in **Figure 5B**.

**Figure 6:**
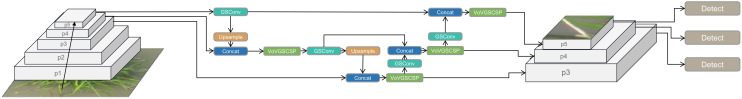
Structural diagram of the neck.

Ultimately, the neck network underwent refinement through the amalgamation of GSConv and VoVGSCSP. These enhancements served to diminish the model’s overall computational overhead, resulting in swifter network operation and reduced information processing time. This harmonious adjustment better balances the trade-off between detection speed and accuracy. The augmented configuration of the neck network is visually presented in the provided **Figure 6**.

#### 2.2.5 Oriented bounding box

The UAV was taken from above, and the target in its images usually appears at any angle. The wild rice leaves are striped and lanceolate in shape, and the bacterial blight is also striped on the leaves. This makes the horizontal bounding box of the original YOLOv8 model overlap too much and inevitably introduces too much background information into the horizontal bounding box of adjacent targets, which not only leads to the phenomenon of missed detection and wrong detection at the same time but also increases the difficulty of extracting features from the network (Zhang et al., 2021). Recently, oriented bounding boxes, which include an angular dimension, have been utilized to represent objects with different orientations (Liao et al., 2018). For instance, Zhang et al. (2023) utilized oriented bounding boxes to detect *Fusarium* head blight (FHB) in wheat and achieved notably high accuracy and robustness in predicting FHB levels. The use of oriented bounding boxes for detecting crop diseases from a UAV perspective has proven to be effective. In light of this, the model proposed in this study adopts oriented bounding box detection. This approach involves angular regression (Ren et al., 2015) and employs the long-edge definition method (Ma et al., 2018) to regress the minimum bounding rectangle of the target. This enhancement is crucial for ensuring reliable and accurate wild rice bacterial blight detection in field conditions.

**Figure 7:**
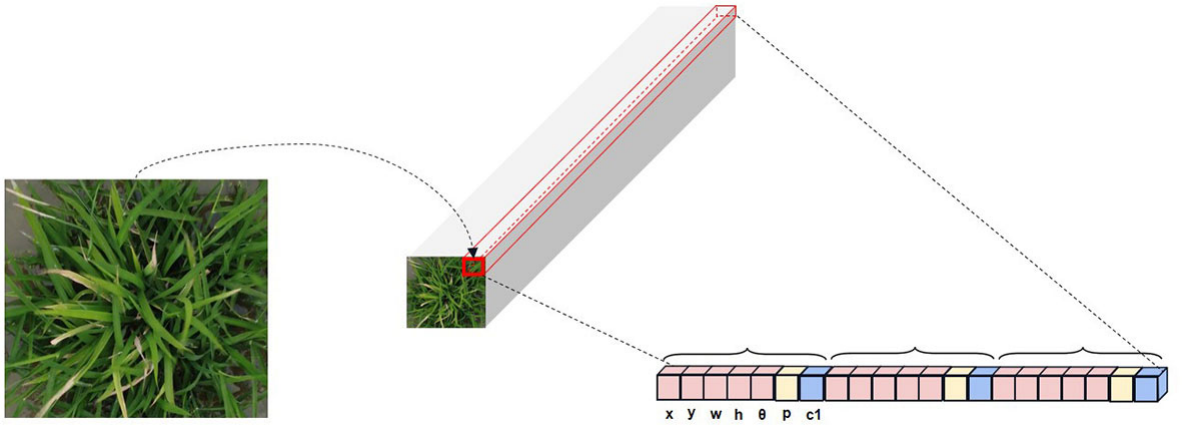
Structural diagram of the head.

**Figure 8:**
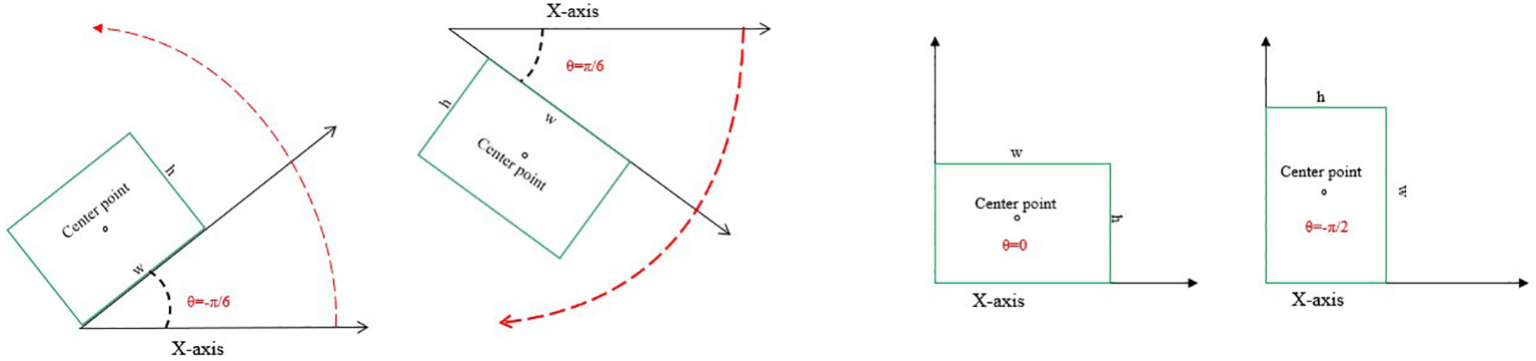
Long-edge definition method.

The horizontal bounding box parameters are expressed as (*x*, *y*, *w*, and *h*). The four parameters indicate the horizontal and vertical coordinates of the center of the horizontal bounding box, width, and height, respectively. The oriented bounding box contains five parameters (*x*, *y*, *w*, *h*, and *θ*), with *θ* indicating the angle of rotation (Li et al., 2020). The structural diagram of the head is shown in **Figure 7**. To prevent duality, the long-edge definition method (**Figure 8**) is used, i.e., *θ* is between −90° and 90°, *w* is the longest edge, its neighbor is *h*, and *θ* represents the range of angles through which the *x*-axis is rotated to *w*. The model proposed in this paper adds a new rotation angle prediction channel to the head structure to implement the detection of the oriented bounding box, with a dimension of 3 × (5 + 1 + *C*), where 3 represents that each grid will be predefined with three predicted boxes of various aspect ratios, 5 represents that each predicted box will predict the parameter (*x*, *y*, *w*, *h*, and *θ*) of the border, 1 is used to determine whether each grid contains the object, and the final *C* parameters is used to determine the type of object each grid contains.

The loss function of the model proposed in this paper consists of three parts: Reg part, Obj part, and Cls part. The Reg part is the regression parameter judgment of the feature points, the Obj part is the judgment of whether the feature points contain objects, and the Cls part is the kind of objects contained in the feature points. In order to avoid the differences between the angular parameter *θ* and other parameters to bring difficulties to the training of the model, the Reg part uses KLD as the loss function (Yang et al., 2021), whose core idea is to convert the oriented bounding box into a two-dimensional Gaussian distribution, i.e. (*x*, *y*, *w*, *h*, and *θ*) into a two-dimensional Gaussian distribution of $N_{\left(\mu ,\text{Σ}\right)}$ , and the conversion is shown in Eq. (10). The KLD between the Gaussian distribution is calculated as the regression, the loss is calculated as shown in Eq. (11), where the subscripts *p* and *t* denote the predicted distribution results and the actual results respectively. The KLD loss function is scale-invariant and can dynamically adjust the gradient weights of the angle parameters according to the aspect ratio of the object, and this self-modulation optimization mechanism effectively promotes the accuracy of oriented bounding box detection. The Obj and Cls parts, on the other hand, adopt a binary cross-entropy loss function to reduce the computational complexity of training.


(10) $$\begin{array}{l} \;\text{μ}={\left(\text{x},\text{y}\right)}^{⊤}\\ \Sigma^{1/2}\&=RAR^{⊤}=\left(\begin{array}{cc} \cos\;\theta & -sin\theta \\ \sin\;\theta & \cos\;\theta \end{array}\right)\left(\begin{array}{cc} \frac{\text{w}}{2} & 0\\ 0 & \frac{\text{h}}{2} \end{array}\right)\left(\begin{array}{cc} \cos\;\theta & \sin\;\theta \\ -\sin\;\theta & \cos\;\theta \end{array}\right)\\ \;=\left(\begin{array}{cc} \frac{\text{w}}{2}cos^{2}\;\theta +\frac{\text{h}}{2}sin^{2}\;\theta & \frac{\text{w }-\text{ h}}{2}\cos\;\theta \sin\;\theta \\ \;\frac{\text{w }-\text{ h}}{2}\cos\;\theta \sin\;\theta & \frac{\text{w}}{2}sin^{2}\;\theta +\frac{\text{h}}{2}cos^{2}\;\theta \end{array}\right) \end{array}$$

(11) $$\begin{array}{c} {\text{D}}_{\text{kl}}\left(N_{{\text{p}}^{\prime }}∥N_{{\text{t}}^{\prime }}\right)=\frac{1}{2}{\left({\text{μ}}_{\text{p}}-{\text{μ}}_{\text{t}}\right)}^{\text{T}}\Sigma_{\text{t}}^{-1}\left(\mu_{p}-\mu_{t}\right)+\frac{1}{2}Tr\left(M^{T}{\left(M^{T}\right)}^{-1}\Sigma_{\text{t}}^{-1}{\text{M}}^{-1}\text{M}\Sigma_{\text{P}}\right)\\ +ln\frac{\left|\Sigma_{\text{t}}\right|}{\Sigma_{\text{P}}}-1\\ {ℒ}_{KLD}=1-\frac{1}{\text{τ }+\text{ f}\left(D\right)},\text{τ}\geq 1 \end{array}$$

## 3 Experiments and analysis of results

### 3.1 Training procedures

The operating environment for this experimental was a Dell tower workstation (Dell, Inc) with an operating system environment of Windows 11, a 12th-Gen Intel^®^ Core™ i5-12500 3.00 GHz processor, 32 G of on-board running memory, a 1-TB solid-state drive, and an NVIDIA GeForce RTX 3080 graphics card with 10 GB of video memory and using GPU-accelerated computing. (Software environment: Python 3.7.16, PyTorch 1.7.0, Torchvision 0.8.2, CUDA 11.0.)

The number of iterations in this experiment was 700, batch_size was set to 2, and Adam was used as the optimizer. The initial learning rate of the models was 1*e*−3, the maximum learning rate was 1*e*−5, the momentum was 0.937, the weight decay was 0, and the input image resolution was 640 × 640. The same training parameters and dataset were used for all models during training.

### 3.2 Performance evaluation

To accurately evaluate the effectiveness of the method proposed in the previous section, this paper uses several evaluation metrics, including precision (*P*), recall (*R*), *F*1 score, mean average precision (mAP), speed (FPS), number of parameters (Params), and GFLOPs. These metrics are utilized to assess the performance of the model in terms of its detection accuracy and efficiency.

The precision refers to the proportion of correctly classified positive samples out of all the samples predicted. It is calculated using the formula presented in Eq. (12).


(12) $$Precision=\frac{TP}{TP+FP}\;$$

Where TP represents the number of true-positive samples (correctly predicted positive samples), and FP represents the number of false-positive samples (negative samples incorrectly predicted as positive).

The recall quantifies the proportion of positive samples that are correctly identified by the model out of the total number of actual positive samples. It is calculated using the Eq. (13).


(13) $$Recall=\frac{TP}{TP+FN}$$

The *F*1 score combines both precision and recall into a single value. The equation for calculating the *F*1 score is shown in Eq. (14).


(14) $$\text{F}1=2\cdot \frac{\text{P}\cdot \text{R}}{\text{P}+\text{R}}$$

The mAP is calculated based on the precision–recall (PR) curve, which only needs to detect a single disease; mAP is equivalent to AP. The equation for calculating mAP is shown in Eq. (15).


(15) $$\text{mAP}=\text{AP}=\int_{0}^{1}P(R)dR$$

The Params reflects the model’s complexity and capacity to learn and represent features. The equation for calculating Params is shown in Eq. (16).


(16) Params =[i·(k·k)·o]+o

Where *i* is the input size, *k* is the convolution kernel size, and *o* is the output size.

Speed is measured in frames per second (FPS). The equation for calculating speed is shown in Eq. (17).


(17) Speed = frames / time

GFLOPS is the speed of the model based on computation costs. The formula for calculating GFLOPS is shown in Eq. (18).


(18) GFLOPS =H·W·params

Where *H* × *W* is the size of the outputted feature map.

### 3.3 Ablation experiment

To offer a more comprehensive understanding of the effectiveness of the proposed enhancement technique applied to the Xoo-YOLO model, a series of ablation experiments were conducted. YOLOv8 was employed as the baseline model for comparison, and the results are detailed in **Table 1**.

(1) Effects of LSKNet: a comparative analysis between YOLOv8 and YOLOv8+LSKNet highlights the efficacy of integrating LSKNet. Notably, the addition of LSKNet leads to a notable enhancement in model accuracy. The metrics mAP@0.5, precision, and recall show improvements of 2.26%, 4.60%, and 4.27%, respectively. This substantiates that LSKNet indeed contributes to improved model performance, attributed to its dynamic adaptation of a large spatial receptive field.
(2) Effects of GSConv: a comparison between YOLOv8 and YOLOv8 + GSConv reveals that the incorporation of GSConv contributes to a reduction in computational costs, with GFLOPS and Params experiencing reductions of 45.89% and 53.15%, respectively. Simultaneously, feature extraction capabilities receive a modest boost, as evidenced by increases of 0.18% in mAP@0.5, 3.80% in precision, and 4.42% in recall. While the GSConv module was integrated into the neck with careful consideration, it was deliberately excluded from the backbone to prevent an excessive presence of GSConv modules. This decision aimed to circumvent the overcomplication of the network architecture, which could hinder spatial information flow and significantly elongate inference times.
(3) Effects of both together: Xoo-YOLO harmoniously amalgamates the strengths of both LSKNet and GSConv. The result is a model with a 45.43% reduction in parameter count, a 51.39% decrease in computational demand, a 10.96% enhancement in precision, a 4.34% improvement in recall, and a noteworthy 7.80% advancement in mAP@0.5 when compared to YOLOv8.

**Table 1:** Comparisons of ablation experiments.

Models	Precision (%)	Recall (%)	mAP@0.5 (%)	GFLOPS (G)	Params (M)
YOLOv8	87.94	75.66	87.15	54.317	24.352
YOLOv8 + LSKNet	90.20	79.93	91.75	54.662	24.786
YOLOv8 + GSConv	88.12	79.46	91.57	29.347	11.404
**YOLOv8 + GSConv + LSKNet (proposed model)**	**97.60**	**80.00**	**94.95**	**29.692**	**11.838**

Bold values represent the model proposed in this paper, namely the Xoo-YOLO model.

Collectively, Xoo-YOLO exemplifies a well-rounded synergy between accuracy enhancement and model lightweightness, thus affirming the significance of our proposed enhancements.

### 3.4 Comparative experiment

**Figure 9:**
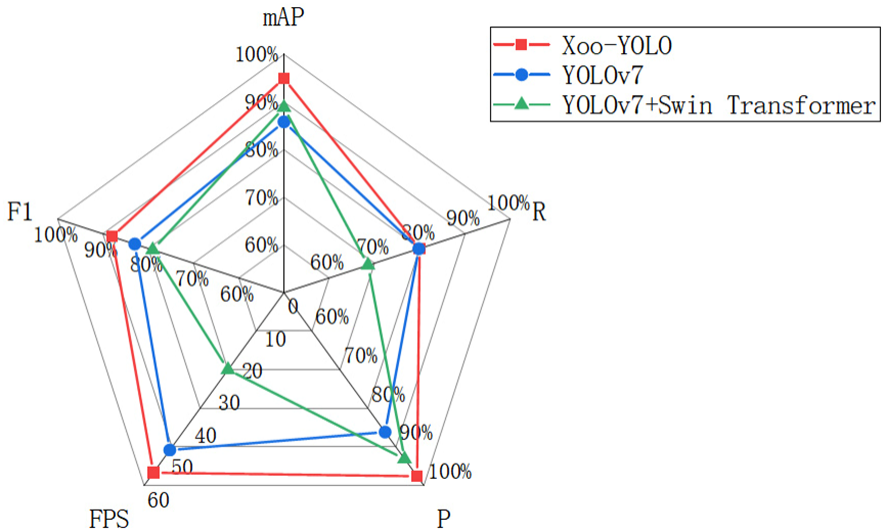
Comparison of detection performance between different models.

To validate the advantages of the model, the wild rice bacterial blight dataset, which includes 450 images in the training set, 150 images in the validation set, and 150 images in the test set, was used to evaluate the model’s performance in terms of precision, recall, *F*1 score, mAP, speed (FPS), Params, and GFLOPs. All experiments were conducted under identical experimental conditions to ensure a fair comparison. Comparative experiments were conducted on the proposed model with the YOLOv7 and YOLOv7-added Swin Transformer module. The comparison results are shown in **Figure 9** and **Table 2**. The detection performance of the three networks is different, and the mAP@0.5 of the Xoo-YOLO proposed in this paper is 94.95%, which is 9.13% and 6.13% higher than the original YOLOv7 and YOLOv7-added Swin Transformer module.

**Table 2:** Comparison of detection performance of different models.

Models	Precision (%)	Recall (%)	*F*1	mAP@0.5 (%)	FPS
YOLOv7	86.15	79.81	0.83	85.82	49
YOLOv7 + Swin Transform	93.13	68.59	0.79	88.82	24
**Proposed model**	**97.6**	**80.00**	**0.88**	**94.95**	**56**

Bold values represent the model proposed in this paper, namely the Xoo-YOLO model.

**Figure 10:**
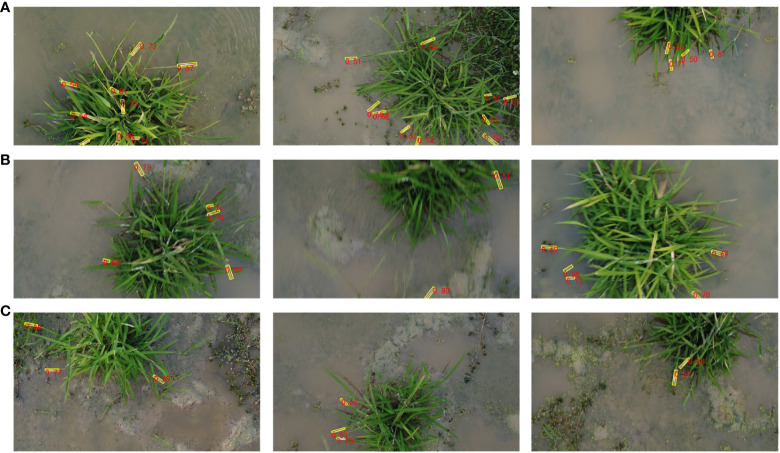
Prediction results of the proposed method. **(A)** Under dense disease conditions. **(B)** Under the conditions of image blurriness generated during the UAV flight collection process. **(C)** Under complex backgrounds such as weeds and debris in the field.

Furthermore, the Xoo-YOLO model outperforms the other models in various metrics. There are two main reasons contributing to this phenomenon. Firstly, in comparison to the ELAN module in YOLOv7, the C2f module in YOLOv8 incorporates a parallel concatenating operation of the bottleneck module, which allows for more branching quadratic links and thus richer gradient flow information and thus possesses enhanced feature extraction and fusion capabilities. Secondly, the LSKNet module dynamically adjusts the spatial receptive field as needed, effectively mitigating instances of false positives and false negatives. These factors collectively reinforce the efficacy of the Xoo-YOLO model in wild rice bacterial blight detection. In order to further verify the performance of the proposed Xoo-YOLO model, we randomly selected some detection results under different environmental conditions from all testing samples, as shown in **Figure 10**.

**Figure 11:**
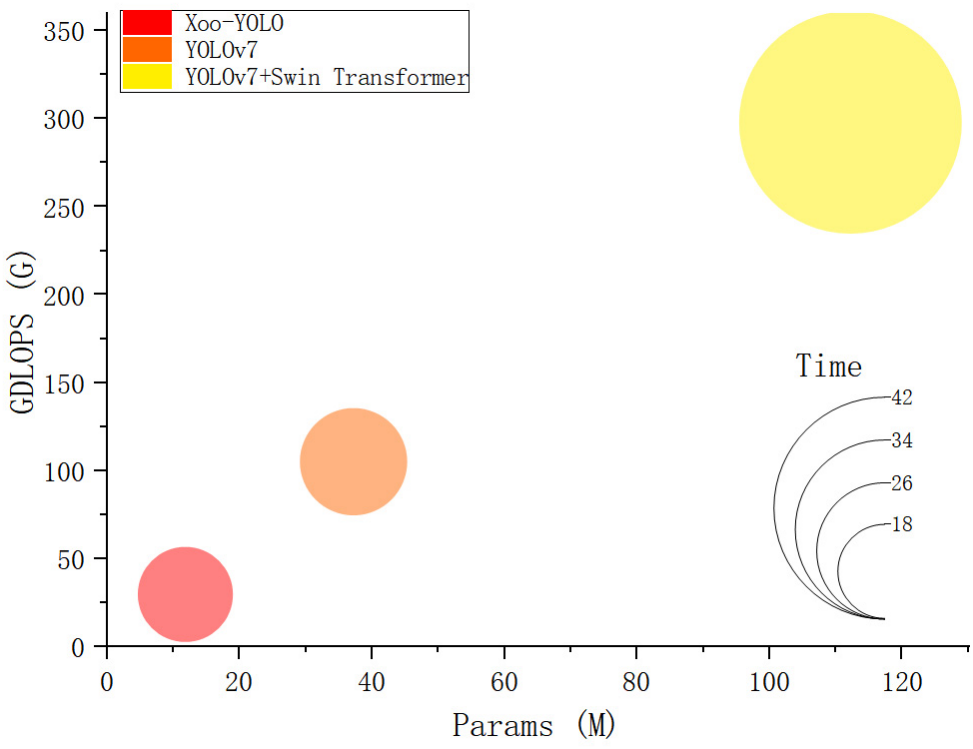
Comparison of different models in terms of computational complexity, parameter, and detection time.

In terms of speed and model size, the Xoo-YOLO model exhibits a significant reduction in parameter count and computational complexity. This reduction is primarily attributed to the introduction of the GSConv module, which has a lower computation intensity. This module effectively accelerates feature fusion while decreasing computational complexity. Additionally, when compared to the YOLOv7 model, the C2f module and SPPF module utilized in YOLOv8 are more lightweight, providing advantages in terms of parameter and computational complexity. Comparative results are presented in **Figure 11**.

To analyze the performance differences between the proposed oriented box detection method and the original horizontal box detection method for wild rice bacterial blight, 750 images were annotated by the horizontal bounding box method and fed into the same network for training. Comparative experiments were conducted on the trained models, and the results are presented in **Table 3**.

**Table 3:** Comparison of the horizontal bounding box and oriented bounding box detection performance.

Models	Precision (%)	Recall (%)	mAP (%)
HBB	73.3	78.5	79.5
**Proposed model**	**97.60**	**80.00**	**94.95**

Bold values represent the model proposed in this paper, namely the Xoo-YOLO model.

**Figure 12:**
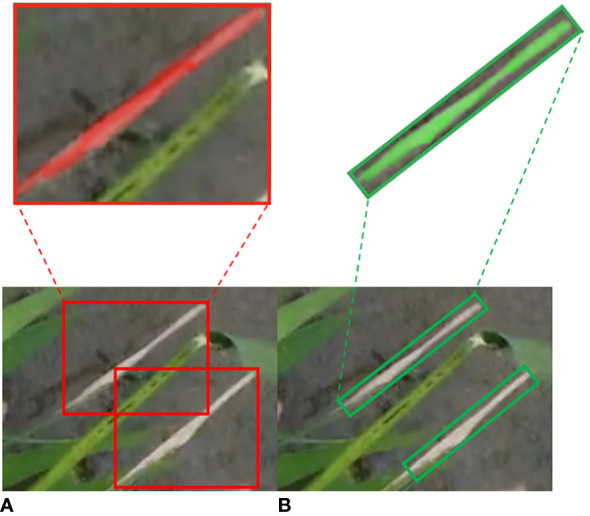
Comparison of detection performance between horizontal bounding boxes (HBB) and oriented bounding boxes (OBB). **(A)** HBB. **(B)** OBB.

Compared to the original method of using horizontal bounding boxes for detection, the model proposed in this study demonstrates improvements in terms of recall, precision, and mAP. When dealing with wild rice bacterial blight cases characterized by large aspect ratios and varying orientations, the utilization of oriented bounding box detection provides a better fit for the diseases. This approach reduces the influence of the background and facilitates more accurate feature extraction. Conversely, employing horizontal bounding boxes for detection can lead to visual disturbances and result in more pronounced instances of missed and false detections. Such an approach is inadequate for addressing the requirements of detecting wild rice bacterial blight from the UAV’s perspective in field conditions. Furthermore, it significantly hampers subsequent research involving disease segmentation and disease resistance identification for wild rice bacterial blight. The comparative results of detection performance using both methods are illustrated in **Figure 12**.

## 4 Discussion

Extensive research on rice bacterial blight detection using deep learning has been conducted. However, prior studies by Haque et al. (2022); Jia et al. (2023); Kumar et al. (2023), and Prasomphan (2023) did not fully consider the complexities of field conditions and the diverse angles at which diseases appear under the UAV viewpoint. This study addresses these specific needs. Moreover, there has been a scarcity of studies involving wild rice bacterial blight detection utilizing deep learning methods. To our knowledge, this is pioneering research to detect wild rice bacterial blight under UAV viewpoints in field settings. The results underscore the considerable potential of the Xoo-YOLO model in disease detection.

The advantages of the Xoo-YOLO model are as follows:

1. Efficiency and speed: Xoo-YOLO boasts lightweight characteristics and enhanced processing speed, making it suitable for deployment on UAV or edge devices.
2. Balance of accuracy and efficiency: The model strikes a harmonious equilibrium between lightweight design and detection accuracy, outperforming other common deep learning models in detection accuracy.
3. Rotated bounding box detection: The Xoo-YOLO model introduces a method for detecting wild rice bacterial blight through oriented bounding boxes, leading to more accurate disease detection and localization. This approach minimizes interference caused by excessive background information under UAV viewpoints, thus establishing a robust foundation for subsequent disease segmentation and measurement efforts.

However, the recall of the Xoo-YOLO model for detecting wild rice bacterial blight stands at 80.0%, suggesting room for improvement. This lower recall could be attributed to factors such as wind interference and motion blur caused by UAV propellers, as well as instances of closely clustered diseases leading to missed detections. To address these, future experiments will explore the use of adversarial generative networks. Additionally, future endeavors should place a priority on including a wide range of wild rice varieties in the research. This approach is essential to ensuring the robustness and generalizability of the proposed method.

While Xoo-YOLO has its limitations, it serves as a valuable technical reference for detecting wild rice bacterial blight in field environments under the UAV viewpoint. The application of the Xoo-YOLO model to an intelligent assessment platform for wild rice diseases holds the promise of validating its reliability.

## 5 Conclusion

Wild rice disease detection is a crucial step in screening and cultivating highly disease-resistant rice varieties. To achieve rapid and accurate detection of bacterial blight in wild rice under natural field conditions, this study establishes a dataset for field-based disease detection. Addressing the unique characteristics of detecting wild rice bacterial blight from UAV viewpoints, this research builds upon the YOLOv8 model, introduces enhancements, and proposes the Xoo-YOLO network architecture. This is achieved by incorporating the LSKNet network in the backbone, integrating the GSConv module in the neck, and adopting oriented bounding box detection. These improvements enable real-time automatic detection of bacterial blight in wild rice under UAV perspectives.

Experimental results reveal that the proposed model achieves an impressive mAP@0.5 of 94.95%. It outperforms comparative models in terms of precision, recall, and *F*1 score. The model demonstrates superior computational complexity, parameter, and detection time with values of 29.692 G, 11.838 M, and 17.78 ms, respectively. These improvements compared to classic object detection models like YOLOv7 and YOLOv8 are significant. The model is well-suited for subsequent research focusing on disease segmentation and disease resistance identification in wild rice bacterial blight.

Considering the requirements of disease resistance identification standards, factors beyond disease detection, such as disease length, need integration with other models like disease segmentation. This study solely focuses on disease detection. However, in future work, we plan to delve into disease segmentation and disease resistance identification to enhance the efficiency of disease resistance gene exploration in wild rice.

## Data availability statement

The raw data supporting the conclusions of this article will be made available by the authors, without undue reservation.

## Author contributions

PP: Conceptualization, Methodology, Project administration, Software, Validation, Writing – original draft, Writing – review & editing. WG: Investigation, Methodology, Validation, Writing – review & editing. XZ: Methodology, Resources, Supervision, Writing – review & editing. LH: Methodology, Resources, Supervision, Writing – review & editing. GZ: Methodology, Resources, Supervision, Writing – review & editing. JZ: Conceptualization, Methodology, Resources, Supervision, Writing – original draft, Writing – review & editing.
